# Antiparasitic Activity of Silver Nanoparticles Synthesized from *Artemisia cina* Aqueous Extract Against *Haemonchus contortus*

**DOI:** 10.3390/pathogens14121251

**Published:** 2025-12-07

**Authors:** Lucero Itzel Hernández Guerrero, Rosa Isabel Higuera-Piedrahita, Héctor Alejandro de la Cruz-Cruz, Jorge Alfredo Cuéllar-Ordaz, María Eugenia López-Arellano, Jocelyn Maza-Lopez, Abraham Méndez-Albores, Alma Vázquez-Durán

**Affiliations:** 1 Facultad de Estudios Superiores Cuautitlán, Universidad Nacional Autónoma de México, Cuautitlán Izcalli 54714, Mexico; luci.hgro@gmail.com (L.I.H.G.); delacruz@unam.mx (H.A.d.l.C.-C.); albores@unam.mx (A.M.-A.); 2 Centro Nacional de Investigación Disciplinaria en Salud Animal e Inocuidad, Instituto Nacional de Investigaciones Forestales, Agrícolas y Pecuarias, Carr. Fed. Cuernavaca-Cuautla 8534, Jiutepec 62550, Mexico; mlopez.arellano@gmail.com (M.E.L.-A.); jomal1993@gmail.com (J.M.-L.)

**Keywords:** silver nanoparticles, *Artemisia cina*, *Haemonchus contortus*, antiparasitic potential

## Abstract

Parasitic infections, particularly those caused by *Haemonchus contortus* (*H. contortus*), severely impact livestock production, with growing resistance to commercial anthelmintics posing a major challenge. Green-synthesized metallic nanoparticles using *Artemisia cina* (*A. cina*), a plant with known anthelmintic and antioxidant properties, represent a promising sustainable alternative for parasite control. In this study, silver nanoparticles (AgNPs) were synthesized using an aqueous extract of *A. cina* to evaluate their anthelmintic activity against infective larvae (L3) of *H. contortus* and their effect on the expression of oxidative stress-related genes. The larval mortality bioassay was conducted in 96-well microtiter plates by incubating L3 larvae with increasing AgNP concentrations for 24 h. To investigate oxidative stress responses, larvae were exposed to sublethal concentrations of AgNPs, *A. cina* aqueous extract, AgNO_3_, and H_2_O_2_. Expression levels of SOD, GPx, and CAT genes were then quantified by RT-qPCR at multiple post-exposure time intervals. The synthesis was optimized by varying parameters such as pH, temperature, and extract volume. The nanoparticles were characterized using UV-Vis spectroscopy, Fourier Transform Infrared Spectroscopy (FTIR), Transmission Electron Microscopy (TEM), Dynamic Light Scattering (DLS), and Electrophoretic Light Scattering (ELS). Overall, synthesis at pH 8 yielded small, spherical, stable, and abundant AgNPs. In vitro assays on L3 larvae showed a mortality rate of 91.33% at the highest AgNP concentration (500 μg/mL), with lethal concentration (LC50 and LC90) values of 4.128 ppm (μg/mL) and 17.993 μg/mL, respectively. Relative expression analyses revealed that AgNPs induced the overexpression of the *SOD* gene, highlighting its role in the oxidative stress response. In contrast, the expression levels of *GPx* and *CAT* genes were markedly downregulated. These results suggest that *SOD* could serve as a potential biomarker of oxidative stress induced by AgNPs in combination with *A. cina* metabolites, influencing the infective stages of *H. contortus*.

## 1. Introduction

*Haemonchus contortus* (*H. contortus*) is one of the most significant nematode parasites affecting sheep globally. It causes substantial economic losses to the sheep industry through reduced productivity, increased mortality, and high treatment costs [1]. However, controlling *H. contortus* has become increasingly challenging due to the widespread emergence of anthelmintic resistance, which has significantly reduced the efficacy of most commercially available anthelmintics [2,3].

In recent years, there has been increasing interest in integrated parasite management (IPM) strategies for controlling *H. contortus*. These approaches combine multiple control measures, including strategic use of anthelmintics, grazing management, genetic selection, and alternative therapies [4,5]. IPM has proven effective in reducing *H. contortus* infections and mitigating the development of anthelmintic resistance in sheep [4,6,7].

However, the long-term sustainability of current control measures remains a major concern. Therefore, there is an increasing need to develop more sustainable and environmentally friendly strategies to manage *H. contortus*. Promising alternatives include the use of plant-derived bioactive compounds, vaccines, and probiotics [8,9]. In addition, selective breeding programs aimed at enhancing genetic resistance to *H. contortus* represent an effective and durable approach to parasite control [10].

Nanotechnology is a rapidly advancing field that has attracted increasing attention for its wide-ranging applications in medicine, electronics, energy, and environmental sciences [11,12]. In veterinary medicine, nanotechnology holds significant promise for transforming disease management in animals [13,14]. The development and application of nanoparticles have opened new opportunities for generating more effective and targeted therapeutic strategies [15]. Owing to their unique physicochemical properties—such as nanoscale size, large surface area, and tunable surface charge—nanoparticles can improve drug delivery, reduce toxicity, and enhance therapeutic efficacy [16]. In particular, the use of nanoparticles for controlling parasitic diseases has shown great promise in improving the delivery and efficacy of anthelmintics, reducing drug resistance, and minimizing environmental contamination [14,17]. Consequently, the integration of nanotechnology into veterinary medicine could substantially improve animal health and productivity while reducing the economic impact of parasitic diseases in the livestock industry [15,18].

The green synthesis of silver nanoparticles (AgNPs) using plant extracts provides a sustainable and eco-friendly alternative to conventional chemical methods. Plant-derived metabolites such as polyphenols, proteins, and terpenoids in plants (found in species like *Artemisia cina*) function as natural reducing and stabilizing agents, enabling the formation of biocompatible AgNPs with potential applications in antimicrobial therapy, drug delivery, and cancer treatment. Key synthesis parameters including pH, temperature, and extract volume critically influence nanoparticle size, stability, and yield. In general, alkaline conditions (pH 8–10) and elevated temperatures promote the formation of smaller and more stable AgNPs [17,19,20].

AgNPs have shown considerable promise in veterinary medicine, particularly against parasitic nematodes such as *H. contortus*. Their antiparasitic action involves the induction of oxidative stress (characterized by *SOD* overexpression and suppression of *GPx* and *CAT* gene activity) along with cuticle damage and increased reactive oxygen species (ROS) generation, ultimately leading to parasite mortality [21]. However, concerns remain regarding their non-selective cytotoxicity, as AgNPs can accumulate in vital organs such as the lungs, liver, and brain potentially disrupting mammalian cell function [11]. Therefore, further research is required to optimize their therapeutic efficacy while minimizing toxicity risks.

*Artemisia cina* (commonly known as Santonica) is a perennial herb belonging to the *Asteraceae* family, traditionally used for the treatment of bacterial, fungal, and parasitic infections owing to its diverse array of bioactive compounds, including sesquiterpenes (such as santonin and artemisinin), flavonoids, and coumarins. Notably, santonin exhibits strong anthelmintic effects against *H. contortus*, significantly reducing parasite burden in lambs within 7–14 days post-treatment [22,23]. The plant’s antioxidant properties are primarily attributed to its polyphenolic constituents, particularly flavonoids, which scavenge reactive oxygen species (ROS), whereas artemisinin—a sesquiterpene lactone—induces parasite death through oxidative stress–mediated mechanisms [24,25].

The host immune response to *H. contortus* is primarily Th2-mediated, characterized by elevated levels of IL-4, IL-5, and IgE/IgG antibodies, along with eosinophil and mast cell infiltration. To evade these immune defenses, the parasite upregulates antioxidant enzymes such as glutathione peroxidase (GPx) to neutralize ROS. However, excessive ROS production—whether induced by pharmacological agents or nanoparticles—can surpass the parasite’s antioxidant capacity, leading to oxidative damage of lipids, proteins, and DNA, and ultimately resulting in parasite death [26]. Based on this understanding of the parasite’s antioxidant defense system, we hypothesized that additional exogenous oxidative stress could surpass its detoxification capacity. In this context, AgNPs constitute a strong source of such stress. Oxidative stress plays a dual role in parasitic infections: while host-derived ROS such as superoxide and hydrogen peroxide (H_2_O_2_) exert antiparasitic effects, *H. contortus* counteracts this stress through antioxidant defenses, including selenium-dependent glutathione peroxidase (GPx), catalase (CAT), and superoxide dismutase (SOD). Notably, exposure to AgNPs induces SOD overexpression in the parasite, suggesting its potential as a biomarker of oxidative stress, whereas GPx and CAT activities are significantly reduced [27,28,29]. This redox imbalance highlights the potential of oxidative stress-inducing agents such as AgNPs in combination with *A. cina* metabolites as targeted strategies for parasite control.

This study aimed to assess how silver nanoparticles synthesized with the aqueous extract of *Artemisia cina* modulate the expression of key oxidative stress–associated genes in infective larvae of *H. contortus*, providing insights into their potential mechanism of antiparasitic action.

## 2. Materials and Methods

### 2.1. Chemicals

Silver nitrate and ammonium hydroxide were purchased from Sigma Chemical Co., Ltd. (St. Louis, MO, USA). All other reagents were of analytical grade, and deionized water was used as a solvent. The primers were synthesized at the Institute of Biotechnology, National Autonomous University of Mexico (UNAM).

### 2.2. Plant Material

*Artemisia cina O. Berg ex Poljakov* (*Asteraceae*) was obtained from Hunab Laboratory (Mexico). The specimen was authenticated by Dr. Alejandro Torres-Montúfar and deposited in the herbarium of the Facultad de Estudios Superiores Cuautitlan (FES-C), Mexico, under voucher No. 11967. The plant was cultivated in soil with a pH of 6.3, at 24 °C, and 80% humidity. Leaves and stems were collected, washed with distilled water to remove dust and impurities, and dried in a forced-air oven at 40 °C. The dried material was milled using an electric plate-style mill type C-11-1 (Glen Mills Inc., Clifton, NJ, USA) and sieved through a 60-mesh screen to obtain particles < 250 µm. The resulting ground material was stored in vacuum-sealed plastic containers at 4 °C until use.

### 2.3. Synthesis and Characterization of Silver Nanoparticles

#### 2.3.1. Preparation of the Aqueous Extract of *A. cina*

To prepare the plant extract, 5 g of *A. cina* powder was mixed with 100 mL of distilled water in an Erlenmeyer flask and heated at 85 °C for 10 min with constant stirring. The mixture was then centrifuged at 3500× *g* for 7 min, and the supernatant was filtered through a 0.22 µm PTFE syringe filter. The resulting extract was stored at 4 °C until further use in nanoparticle synthesis.

#### 2.3.2. Synthesis of Silver Nanoparticles

AgNPs were first synthesized at 90 °C using 0.01 M AgNO_3_ and a 9:1 volume ratio of AgNO_3_ to *A. cina* extract. Reaction kinetics were monitored by UV–Vis spectroscopy over a 0–100 min period, during which aliquots were collected at predetermined intervals to track the temporal evolution of the surface plasmon resonance (SPR) band. This allowed identification of the nucleation onset and determination of the time required for complete AgNPs formation. Subsequently, to establish the optimal synthesis conditions, a systematic optimization study was performed by varying three key parameters: reaction temperature (30, 60, and 90 °C), pH (6, 7, 8, and 9), and the volume of *A. cina* aqueous extract (9:1, 8:2, and 7:3 ratios of AgNO_3_ to extract). Based on preliminary optimization results, a standardized synthesis protocol was established by mixing 8 mL of aqueous AgNO_3_ solution with 2 mL of *A. cina* extract in a 50 mL glass beaker, resulting in a final silver nitrate concentration of 0.01 M. The pH of the reaction mixture was adjusted to 8 using 29% ammonium hydroxide. The solution was then incubated in the dark at room temperature under continuous stirring to minimize photoactivation of AgNO_3_. The formation of AgNPs was initially confirmed visually by a characteristic color change from colorless to yellowish-brown, characteristic of nanoparticle formation. After synthesis, the AgNPs suspension was purified by three centrifugation cycles (7000 rpm, 7 min, 25 °C) to remove excess Ag^+^ ions and unbound plant metabolites. The pellet was resuspended in deionized water after each cycle. Purified AgNPs, which retained their bio-organic capping layer, were used for all subsequent characterization and biological assays. This optimized and controlled synthesis protocol provides consistent and efficient conditions for the reproducible production of silver nanoparticles, suitable for subsequent physicochemical and biological characterization. All experiments were conducted in triplicate to ensure reproducibility and statistical validity.

#### 2.3.3. Characterization

The reduction of silver ions was monitored by UV-Vis spectroscopy using a Cary 8454 UV-Vis Diode Array System (Agilent Technologies, Santa Clara, CA, USA). Absorbance spectrum was collected from 0 to 100 min, starting immediately after the addition of the *A. cina* extract. Measurements were performed in the 200–1000 nm range, with samples diluted tenfold prior to analysis. A characteristic absorbance peak at ~431 nm confirmed the formation of AgNPs. The zeta potential, size distribution, and polydispersity index (PDI) of the AgNPs were determined using a ZetaSizer Pro (Malvern Instruments, Worcestershire, UK) via electrophoretic light scattering (ELS) and dynamic light scattering (DLS), respectively. The morphology and size of the AgNPs were characterized by transmission electron microscopy (TEM). A 25 µL aliquot of the AgNPs suspension was deposited onto a carbon-coated copper grid and allowed to dry at room temperature. Images were acquired using a JEOL transmission electron microscope (JEOL USA, Inc., Peabody, MA, USA) operated at an accelerating voltage of 80 kV. Particle size measurements were performed with the Analyze Particles routine in ImageJ 1.x, without applying separation methods or constraints. The corresponding size distribution histogram was generated by measuring 200 individual particles. Fourier Transform Infrared Spectroscopy (FTIR) was employed to identify the functional groups in the aqueous extract and on the surface of AgNPs. Spectra were acquired using a Frontier SP800 FTIR-NIR/MIR spectrometer (Perkin-Elmer, Waltham, MA, USA) accessorized with an ATR accessory featuring a diamond crystal. Measurements were carried out in transmittance mode with a spectral resolution of 4 cm^−1^.

### 2.4. Haemonchus Contortus Larvae Obtaining

Larvae of *H. contortus* were obtained by experimentally infecting a four-month-old, parasite-free male lamb (30 kg) with 5000 infective larvae from the FESC strain. Following a 20-day pre-patent period, fecal samples were collected directly from the animal’s rectum. Infective larvae (L_3_ stage) were recovered from fecal cultures using a modified Corticelli-Lai technique after a 14-day incubation period at 24 °C [30]. The donor lamb was maintained under controlled conditions in accordance with animal welfare principles and the Norma Oficial Mexicana DOF 07-06-2012, ensuring minimal discomfort and adherence to ethical standards.

### 2.5. Experimental Design

#### Larval Mortality

Larval damage assessment was performed following the methodology described by Shepherd et al. [31] Experiments were conducted in 96-well microtiter plates, using four wells per treatment in triplicate. AgNPs were tested at concentrations of 500, 250, 125, 62.5, 31.25, 15.6, 7.8, 3.9, and 1.95 ppm. The experimental design included a negative control (distilled water), a positive control (ivermectin, 0.5 mg/mL), an aqueous extract control (*A. cina* extract alone), and a AgNO_3_ control (500 ppm). Approximately 100 *H. contortus* L3 larvae were added to each well containing the respective treatments and incubated in a humid chamber at room temperature for 24 h. After incubation, the entire volume of each well was examined under a light microscope, and live and dead larvae were counted. The larval mortality percentage was calculated using the following equation:

$$\%Mortality=\frac{Number\;of\;dead\;larvae}{Total\;number\;of\;larvae}*100$$

### 2.6. Statistical Analysis

The percentage data for larval mortality were square root–transformed prior to statistical analysis to normalize variance. The transformed data were analyzed using a one-way analysis of variance (ANOVA) under a completely randomized design. Mean comparisons were performed using Tukey’s test at a significance level of *p* < 0.05. The median and 90% lethal concentrations (LC_50_ and LC_90_) were estimated using the PROBIT procedure implemented in the SAS statistical software package 2.0 (SAS Institute Inc., Cary, NC, USA).

### 2.7. Relative Expression of Oxidative Stress Genes

For the evaluation of relative gene expression associated with oxidative stress, the enzymes glutathione peroxidase (GPx), catalase (CAT), and superoxide dismutase (SOD) were analyzed using the methodology described by Higuera-Piedrahita et al. [25] and Maza-López et al. [32,33], with modifications adapted for nanoparticle exposure experiments. Experiments were performed in six-well plates, with three wells assigned per treatment and conducted in triplicate. The experimental design included the following groups: Group 1 (G1): AgNPs at 0.48 ppm; Group 2 (G2): hydrogen peroxide (H_2_O_2_) at 0.078%; Group 3 (G3): silver nitrate at 0.48 ppm; Group 4 (G4): negative control with distilled water; and Group 5 (G5): aqueous extract of *A. cina* at 2500 ppm. All treatments were incubated at room temperature for 30 min, 2, 4, and 8 h [34,35].

### 2.8. Total RNA Extraction

Following incubation, total RNA was extracted from 20,000 infective L3 larvae using the methodology described by Reyes-Guerrero et al. [36]. Larvae were pelleted by centrifugation at 5000 rpm for 5 min at 4 °C, washed with a 40% sucrose solution and sheathed using 0.187% sodium hypochlorite. Each pellet was then resuspended in 500 µL of TRIzol^®^ reagent (Invitrogen, Carlsbad, CA, USA) and stored at 4 °C for 24 h. RNA extraction was facilitated by adding 1 mm zirconium beads to the TRIzol-treated samples, followed by homogenization for 40 s at 400 rpm using a microtube homogenizer (BeadBug D1030, Benchmark Scientific, Sayreville, NJ, USA). After confirming complete larval disruption, the zirconium beads were removed, and 200 µL chloroform (J.T. Baker™, Mexico City, Mexico) was added to each sample. The mixture was gently inverted, incubated for 5 min, and centrifuged at 12,000 rpm for 15 min at 4 °C. The resulting aqueous phase was carefully transferred to a new microtube, mixed with 500 µL cold isopropanol, incubated on ice for 10 min, and centrifuged again under the same conditions. The RNA pellet obtained was washed with 75% ethanol (Hycel, Zapopan, Mexico), centrifuged at 7500 rpm for 5 min at 4 °C, air-dried, and resuspended in 50 µL nuclease-free water (Thermo Scientific™, Waltham, MA, USA). RNA concentration and purity (A260/280) were determined using a NanoPhotometer^®^ NP80 (IMPLEN, Westlake Village, CA, USA).

### 2.9. Reverse Transcription

mRNA reverse transcription (RT) was performed using the ImProm-II™ Reverse Transcription System (Promega, Madison, WI, USA), following the protocol described by Reyes-Guerrero et al. (2020). A total of 300 ng mRNA was used for cDNA synthesis. Gene sequences corresponding to *H. contortus GPx*, *CAT*, and *SOD*, as well as the constitutive 18S ribosomal RNA (rRNA) gene, were obtained from NCBI database (accession: SUB13959139; Maza-López et al., 2024 [33]). All primers were synthesized at the Institute of Biotechnology, UNAM (Cuernavaca, Mexico). Nucleic acid purity (A260/280 ratio: 1.8–2.0) and concentration were confirmed using a NanoPhotometer^®^ (IMPLEN, USA). Primer sequences are listed in Table 1.

### 2.10. Real-Time PCR

The relative expression levels of ROS- associated genes (*GPx*, *CAT*, *SOD*) and the reference gene (18S rRNA) were quantified by RT-qPCR following the manufacturer’s protocol, using a cDNA input of 1500 ng [33]. Reactions were performed in 0.2 mL tubes with a final volume of 20 μL per sample and analyzed in six technical replicates to ensure data reliability. The reaction mixture contained SYBR Green master mix, forward and reverse primers (20 μM each), cDNA template, and nuclease-free water. For each gene and time point, three independent biological replicates were performed. The amplification program consisted of an initial denaturation step at 95 °C for 5 min, followed by 30 cycles of denaturation (95 °C, 15 s), annealing (63 °C, 20 s), and extension (72 °C, 20 s), concluding with a melting curve analysis from 65 to 95 °C. Fluorescence detection was performed using a LightCycler^®^ 96 System (Roche Diagnostics, Indianapolis, IN, USA).

### 2.11. Statistical Analysis of Gene Expression

Gene expression data were analyzed using Qiagen’s GeneGlobe Data Analysis Center (https://geneglobe.qiagen.com/us/analyze/, accessed on 15 August 2024). Relative quantification was performed using the comparative CT (∆∆CT) method: CT values of target genes (GPx, CAT, SOD) were normalized to the reference gene (18S rRNA) to obtain ∆CT values, which were then compared between treatment and control groups to calculate ∆∆CT values. Fold-changes in gene expression were determined as 2^−∆∆CT^. Statistical significance (*p* < 0.05) was assessed using Student’s t-test to compare mean ∆CT values between groups.

## 3. Results

**Figure 1:**
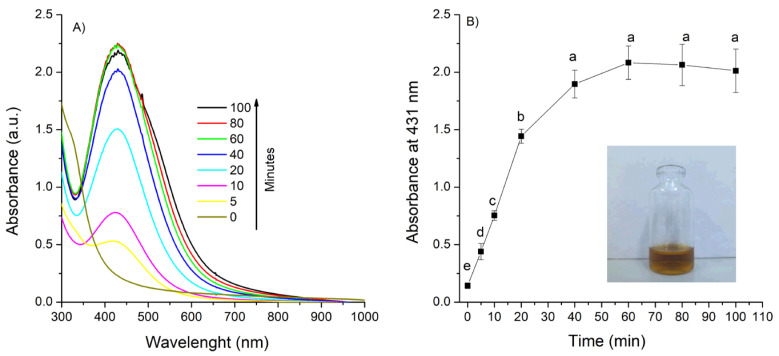
UV–Vis spectra of AgNPs formation as a function of time (**A**,**B**) and time evolution of the absorbance at λ = 431 nm, using 0.01 M of silver nitrate, 1 mL of extract and 90 °C temperature. Means sharing the same letter are not significantly different (*p* > 0.05).

The synthesis of AgNPs using the aqueous extract of *A. cina* was successfully achieved. A distinct color change in the reaction mixture from colorless to yellowish-brown (Figure 1, profile B, inset) provided the first visual indication of *Ag*^+^ ion reduction to metallic silver (*Ag*^0^). The formation and stability of the AgNPs were further monitored by UV–Vis spectroscopy, which showed a characteristic surface plasmon resonance (SPR) band around 431 nm. Figure 1 (profile A) shows the UV-Vis absorbance spectra of the AgNPs solution synthesized with 1 mL *A. cina* aqueous extract and 0.01 M of AgNO_3_, monitored from 0 to 100 min. As the reaction progressed, the spectrum gradually intensified and developed the well-defined SPR, associated with the formation of well-dispersed AgNPs. The evolution and progressive intensification of this peak over time reflects the nucleation and growth stages of nanoparticle formation, confirming the successful reduction of silver ions by the *A. cina* extract into colloidal AgNPs. Figure 1 (profile B) presents the absorbance intensity at 431 as a function of reaction time. The curve shows a rapid increase during the first 40 min, followed by a plateau between 60 and 100 min, confirming that the reaction reaches completion.

**Figure 2:**
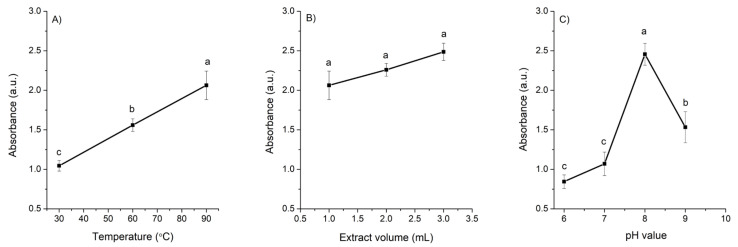
Effect of (**A**) temperature, (**B**) extract volume, and (**C**) pH on the absorbance of AgNPs at 431 nm. Data represent mean ± standard deviation (*n* = 3). Means sharing the same letter are not significantly different (*p* > 0.05).

Thus, a reaction time of 80 min was selected for subsequent experiments, and the effects of the remaining synthesis parameters were then evaluated:

Temperature: The intensity of the SPR band significantly increased with rising reaction temperature (30 °C, 60 °C, and 90 °C). Nanoparticles synthesized at 30 °C exhibited a broader SPR band, indicating larger particle size or partial aggregation, whereas those synthesized at higher temperatures showed sharper and more dined peaks, consistent with the formation of smaller, more monodisperse particles (Figure 2, profile A).

Extract volume: Increasing the volume of the *A. cina* extract in the reaction mixture (from a 9:1 to a 7:3 ratio of AgNO_3_ to extract) resulted in a slight increase in the SPR absorption maximum; however, none of the three volumes evaluated produced a significant effect on the overall spectral profile (Figure 2, profile B). From these results the 8:2 ratio of AgNO_3_ to extract was selected for the subsequent pH evaluation.

pH: In general, the synthesis of AgNPs was more favorable under alkaline conditions. The SPR intensity was highest at pH 8 (2.45 a.u.), where sharper and more defined absorption peaks were observed, indicating the formation of smaller and more uniformly distributed nanoparticles compared with those produced under neutral or slightly acidic conditions (Figure 2, profile C). Because the reaction proceeded instantaneously at all pH values tested, the synthesis was carried out at the lowest temperature evaluated (30 °C) to determine whether heating was necessary. The results confirmed that elevated temperatures were not required for nanoparticle formation; consequently, 30 °C was selected as the optimal condition to ensure a more energy-efficient and environmentally friendly synthesis.

**Figure 3:**
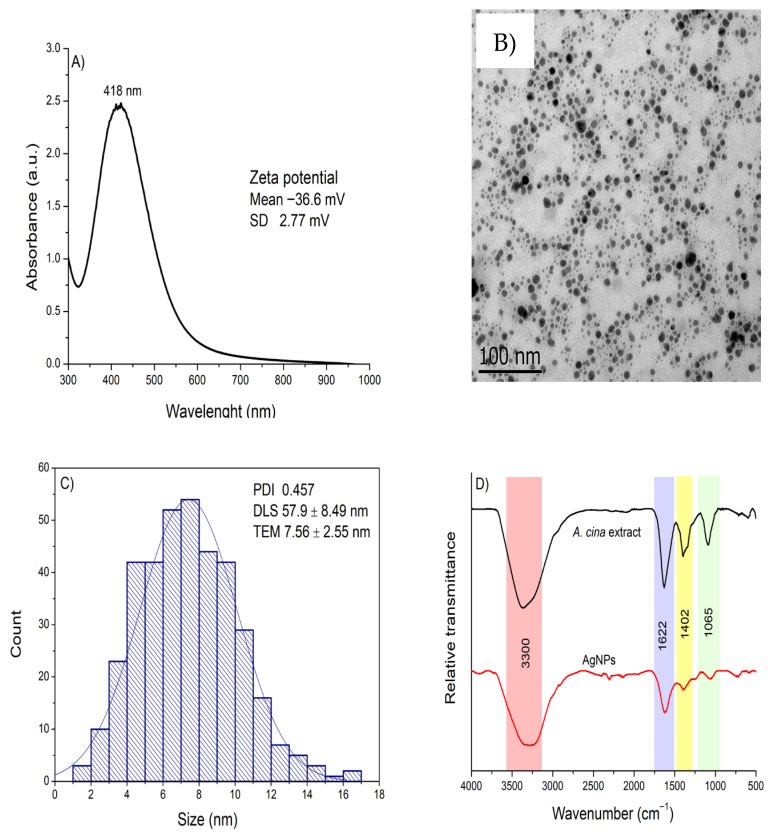
(**A**) UV–Vis absorption spectrum with the zeta potential value shown as an inset; (**B**) transmission electron microscopy image; (**C**) size distribution determined from TEM measurements, with the PDI and hydrodynamic diameter displayed as an inset; and (**D**) FTIR spectra of the *A. cina* aqueous extract and the synthesized AgNPs, obtained under optimal conditions (0.01 M AgNO_3_, 30 °C, an 8:2 ratio of AgNO_3_ to extract, and pH 8).

Based on the optimal SPR characteristics (high intensity and a narrow peak indicative of smaller particle size), along with the consideration of energy efficiency, the synthesis conditions of 30 °C, an 8:2 ratio of AgNO_3_ to extract, and pH 8 were selected as optimal for the adequate synthesis of AgNPs. These AgNPs were further characterized using UV-Vis, ELS, DLS, TEM, and FTIR (Figure 3). Panel A shows the UV–Vis absorption spectrum, which exhibits a sharp and pronounced SPR peak centered at 418 nm, indicative of well-formed metallic nanoparticles with a narrow size distribution. The inset displays the zeta potential (−36.6 ± 2.77 mV), confirming good colloidal stability, as this high negative surface charge promotes strong electrostatic repulsion and prevents particle aggregation. Panel B presents a representative TEM image showing predominantly quasi-spherical nanoparticles, while Panel C reports an average core size of 7.6 ± 2.55 nm derived from TEM measurements. Additionally, DLS analysis (inset of Panel C) revealed an average hydrodynamic diameter of 57.9 ± 8.49 nm and a PDI of 0.457, indicating a moderately homogeneous size distribution. Finally, Panel D shows the FTIR spectra comparing the *A. cina* extract with the synthesized AgNPs. The aqueous extract exhibited four main absorption bands at 3381, 1621, 1387, and 1090 cm^−1^, corresponding to O–H and N–H stretching vibrations, C=C stretching, and C–O stretching vibrations, respectively. In contrast, the AgNP spectrum displayed noticeable shifts and decreased intensity in three of these characteristic bands, indicating that the extract’s biomolecules participated in the reduction and stabilization of the nanoparticles. These spectral changes confirm that hydroxyl and carbonyl groups from polyphenolic compounds served as the primary functional groups responsible for reducing silver ions and capping the resulting nanoparticles.

Overall, the physicochemical characterization confirmed the successful synthesis of stable, predominantly quasi-spherical AgNPs using *A. cina* extract. The nanoparticles exhibited strong SPR absorption, characteristic FTIR bands associated with polyphenolic functional groups, a moderate hydrodynamic size with good colloidal stability, and a small metallic core observed by TEM. These results validate the effectiveness of *A. cina* biomolecules in reducing and stabilizing silver ions, providing a reliable foundation for subsequent antiparasitic activity evaluations.

### 3.1. Larval Mortality

Silver nanoparticles synthesized using the aqueous extract of *A. cina* demonstrated strong larvicidal activity against *H. contortus* L3 larvae, achieving mortality rates of 91.33%, 91.33%, 91.33%, 90.145%, 83.9%, 77.16%, and 75.29% at concentrations of 500, 250, 125, 62.5, 31.25, 15.6, and 7.8 ppm, respectively. A clear concentration-dependent response was observed, with larvae exhibiting increased motility and coiling behavior at lower AgNP concentrations. The aqueous extract of *A. cina* alone showed limited larvicidal activity (21.54% mortality), whereas the negative control (distilled water) produced no lethal effects, with larvae remaining fully motile. In contrast, AgNO_3_ solution showed 83.54% efficacy, comparable to the positive control ivermectin (88.83%). The reduced larval motility observed after 24 h at this high concentration may indicate decreased susceptibility to ivermectin in this *H. contortus* field isolate, a characteristic commonly associated with emerging anthelmintic tolerance. This finding warrants further investigation (Table 2). Furthermore, the 50% lethal concentration 50 (LC50) and the 90% lethal concentration (LC90) of the AgNPs, determined by PROBIT analysis, were 4.128 ppm and 17.993 ppm, respectively.

### 3.2. Gene Expression

The results indicated that, in the AgNPs-treated group, all genes were downregulated at both the 2 and 4 h time points, consistent with previous GPx assays, with statistically significant reductions observed at both intervals. In the hydrogen peroxide group (Table 3), substantial overexpression of the SOD gene was detected at 2 h relative to both the control and the reference gene, whereas CAT remained downregulated and GPx expression returned to baseline. Moreover, by 4 h, all genes in the hydrogen peroxide group were downregulated, with only SOD maintaining statistical significance. In the silver nitrate group (Table 4), a marked upregulation of SOD was observed at 2 h, showing a 29.24-fold increase relative to the reference gene compared to controls, while GPx and CAT were downregulated, with CAT reaching statistical significance. At 4 h, all target genes were completely suppressed, although *SOD* continued to exhibit statistically significant expression. The SOD gene showed significant overexpression at 30 min and again at 8 h post-exposure, consistent with the data from the AgNPs treatment group, as presented in Table 6. In contrast, GPx and CAT were significantly downregulated at 2h. By 4 h post-exposure, the expression levels of all three genes returned to baseline or showed a non-significant reduction compared to the control.

Exposure to hydrogen peroxide (H_2_O_2_, 0.078%), a validated inducer of oxidative stress, resulted in strong SOD overexpression at 2 h (49.64-fold, *p* < 0.05), preceded by significant downregulation at 30 min and 4 h. The GPx and CAT genes were significantly downregulated at most time points (Table 3). In the AgNO_3_ treatment group (0.48 ppm), early SOD upregulation was detected at 30 min (2-fold, *p* < 0.05), along with marked CAT overexpression at 30 min (9.68-fold, *p* < 0.05). Thereafter, SOD, GPx, and CAT expression levels were significantly reduced at 2 h and 4 h (Table 4). The aqueous extract of *A. cina* (2500 ppm) induced significant CAT overexpression at 30 min (3.02-fold, *p* < 0.05), followed by GPx and CAT downregulation at 2 h, and by 8 h, all three target genes were significantly suppressed (Table 5). In the AgNPs group (0.48 ppm), the most notable responses included substantial GPx and CAT downregulation at 2 h, while significant SOD overexpression was observed at 8 h post-exposure (2.67-fold, *p* < 0.05) (Table 6).

The results revealed a differential response of the SOD gene across various treatments in *H. contortus* L3 larvae. Hydrogen peroxide, used as a positive control, induced significant SOD overexpression at 2 h (49.64-fold change), representing the highest upregulation level observed, followed by a sharp decline at later time points, indicative of severe early-stage cellular stress. In contrast, GPx and CAT genes remained either at baseline levels or were downregulated. In the AgNPs-treated group (0.48 ppm, Table 6), SOD exhibited an immediate and sustained response, with overexpression at both 30 min and 8 h post-exposure, suggesting its key role in cellular defense. AgNO_3_, used as comparative control, demonstrated similar temporal dynamics, with SOD upregulated at 30 min and 2 h, followed by downregulation at 4 and 8 h. However, the response was less pronounced than with the *A. cina*-synthesized AgNPs, suggesting that bioactive components from the plant extract contributed significantly to the late-stage (8 h) cellular effects.

The aqueous *A. cina* extract (Table 5) (2500 ppm) elicited distinct activation patterns, with significant CAT expression at 30 min and SOD overexpression at 2 h, which persisted through 4 h, while GPx showed delayed upregulation at 4 h. Notably, despite the substantially lower concentration of AgNPs compared to the crude extract, both treatments induced rapid and sustained SOD overexpression, reflecting a strong cellular defensive response against oxidative stress. Within the context of induced cellular stress, SOD emerged as a key molecular marker, demonstrating significant activity at both 30 min and 8 h post-AgNPs exposure, underscoring its critical protective role against oxidative stress in *H. contortus*. In contrast, CAT and GPx exhibited more conservative regulation patterns, indicating a differential involvement in the parasite’s antioxidant defense system.

## 4. Discussion

The green synthesis of silver nanoparticles (AgNPs) using the aqueous extract of *A. cina* provides an important approach to mitigating concerns related to non-selective cytotoxicity. The plant-derived metabolites that adsorb onto the nanoparticle surface not only enhance colloidal stability, but may also influence nano–bio interactions, potentially modulating their affinity and selectivity toward the parasite. Although this study shows strong in vitro anthelmintic efficacy at low concentrations, comprehensive safety validation will require cytotoxicity assessment in mammalian cell lines and confirmation in in vivo models. These evaluations will be essential to optimize the therapeutic window and define the safe operational range of the synthesized AgNPs, ultimately supporting the rational design of a parasite-selective nanotherapeutic strategy.

The ability of plants to reduce metal ions is often attributed to a phenomenon known as bioaccumulation, which enables them to detoxify excess metals. When exposed to elevated concentrations of metal ions, plants rapidly absorb and metabolize these ions to mitigate their toxic effects. This exposure triggers excessive production of ROS, leading to oxidative stress and cellular damage. To counteract this, plants activate metal-chelating mechanisms and enzymatic antioxidant systems that facilitate detoxification. Furthermore, secondary metabolites such as phenolic compounds play a crucial role in maintaining ROS homeostasis, thereby protecting plant cells from oxidative damage [13,14,37].

A similar mechanism is proposed to occur during plant-mediated nanoparticle synthesis. When metal salts dissociate into cations and anions, the metal cations undergo reduction and subsequently interact with hydroxyl groups, promoting the nucleation and growth of metal nanocrystals. These crystals develop along specific planes until the capping or stabilizing agents present in the plant extract become activated, binding to the nanoparticle surface and halting further crystal growth. This process results in the formation of well-defined nanoparticles. The abundance of reducing and stabilizing phytochemicals—such as polyphenols, flavonoids, terpenoids, and proteins—plays a crucial role in preventing nanoparticle aggregation and favors the formation of smaller, more stable particles [13,19].

The antiparasitic activity of *A. cina* has previously been evaluated using n-hexane and ethyl acetate extracts against *H. contortus* [9]. However, the concentrations reported in that study (1 and 2 mg/mL) were considerably higher compared to the present results obtained with AgNPs synthesized from the aqueous extract of *A. cina*, which exhibited markedly greater potency, with LC_50_ = 4.128 ppm and LC_90_ = 17.993 ppm. Compared to alcoholic extracts, aqueous extracts of *Artemisia* spp. have exhibited lower anthelmintic activity against ovine nematodes in previous studies [35]. This reduced efficacy may be attributed to the fact that approximately 70% of the bioactive compounds in plants are hydrophobic, limiting their solubility and availability in aqueous media.

The results of this study demonstrated that AgNPs synthesized using the aqueous extract of *A. cina* exhibited a significantly higher larval mortality rate compared to the extract alone. This trend aligns with previous reports indicating that metallic nanoparticles synthesized with aqueous plant extracts achieve superior biological efficacy and yield compared to the crude aqueous extracts, likely due to the enhanced bioavailability and stability provided by the nanostructured form [12,13,14,17]. This can be explained by the fact that polar extracts such as methanolic or aqueous extracts are rich in polyphenolic compounds with strong antioxidant properties, which facilitate metal ion reduction and subsequent nanoparticle nucleation and growth [22]. Among the most commonly identified metabolites in aqueous extracts are alkaloids, phenols, flavonoids, and saponins, all of which can participate in the bioreduction and stabilization processes during AgNPs synthesis [20,38]. In contrast, AgNPs synthesized through these extracts are typically enriched with flavonoids, polyphenols (quercetin, caemferol, and luteolin), alkaloids, and terpenoids, reflecting the contribution of these biomolecules as reducing and capping agents that determine the physicochemical properties of the nanoparticles [39].

A comparative analysis of the FTIR spectra between the medicinal plant extract and the biosynthesized AgNPs provides valuable insights into the functional groups involved in nanoparticle surface coating and stabilization. The FTIR spectrum showed prominent bands at 3295, 1621, 1397, and 1060 cm^−1^ in the synthesized AgNPs. These absorption bands correspond to the O–H, N-H, C=C, and C-O vibrations due to the presence of alcoholic, aromatic, and phenolic compounds, which are likely responsible for the reduction of silver ions and subsequent capping of the nanoparticles, thus contributing to their stability. [12,15]. The most relevant functional groups present in plant-derived compounds are hydroxyl and carbonyl groups, which play a crucial role in the reduction of metal ions and stabilization of the resulting nanoparticles. These groups enable plant metabolites to act simultaneously as reducing and capping agents during nanoparticle formation. FTIR studies of *Artemisia sieberi* conducted by Al-Otibi et al. [36] revealed that the plant is rich in amines and carboxyl-containing functional groups, such as alcohols, phenols, and alkanes. The aqueous extract of its leaves exhibited characteristic bands corresponding to hydroxyl, amide, and amine groups, findings that are consistent with the results obtained in the present study. Similarly, in the case of *A. cina*, the aqueous extracts are predominantly composed of polyphenolic compounds, including flavonoids, flavonoid glycosides, and coumarins, along with sesquiterpenes, which are recognized as the principal bioactive components characteristic of this genus. It has been suggested that the size and morphology of nanoparticles are influenced by the interactions between the biomolecules present in plant extracts and metal ions, as each plant species varies in the composition and concentration of these biomolecules. Such differences may partly account for the morphological diversity observed among biosynthesized nanostructures [37]. Moreover, variations in synthesis parameters, including temperature, extract volume, and pH can also contribute to differences in NP shape and size [20,40].

TEM analysis revealed that the AgNPs synthesized using the aqueous extract of *A. cina* exhibited a quasi-spherical morphology. Another authors reported [38], that who synthesized AgNPs with *A. annua* extract and obtained spherical nanoparticles ranging from 20 to 90 nm. The shape and size of metallic nanoparticles is a key determinant of their physical, chemical, optical, and electronic properties. In this study, the spherical morphology of the synthesized AgNPs was further supported by the UV–Vis absorption spectrum, which exhibited a single symmetric SPR band, consistent with the TEM observations.

The size of the AgNPs, as determined by TEM, was 7.56 nm, whereas DLS analysis indicated an average hydrodynamic diameter of 57.9 nm. These differences arise from the distinct principles underlying each technique. It is important to note that nanoparticles are three-dimensional entities, and even when they appear spherical and homogeneous, the measured dimensions may not perfectly reflect their true physical size, depending on the analytical method used. Previous studies have demonstrated that smaller, spherical nanoparticles exhibit enhanced antibacterial activity, attributed to their larger surface area-to-volume ratio and greater ability to penetrate microbial membranes, leading to membrane disruption, increased permeability, and cell death [36]. Moreover, spherical nanoparticles tend to be less toxic than dendritic forms and more effective than triangular ones. Nonetheless, the precise mechanisms by which nanoparticle morphology influences antibacterial efficacy remain not fully understood [36,38]. The results showed that the SPR intensity increased with longer reaction times, consistent with the findings reported by Agustina et al. [41]. It is well established that broader SPR peaks are typically associated with the presence of larger or more polydisperse nanoparticles. Thus, the broader SPR band observed for the AgNPs synthesized at 30 °C suggests slower nucleation and growth dynamics, which may have favored the formation of comparatively larger particles in this study. Conversely, narrower peaks at shorter wavelengths are typically associated with the formation of smaller, well-dispersed nanoparticles.

The nanoparticles synthesized at pH 8 exhibited the highest absorbance, indicating optimal nanoparticle formation compared to other tested pH values. This condition also proved more efficient in terms of reaction time and energy consumption than other evaluated variables such as temperature and extract volume. Similarly, another authors [40] reported that the extract of *Curcuma longa* produced a significantly greater yield of AgNPs under alkaline conditions. Consistent results were also observed [38], with synthesized AgNPs using aqueous extracts of *A. annua*, with nanoparticle formation occurring at pH 7 and 9, while no nanoparticles were detected at pH 3 or 5. It is noteworthy that these authors did not evaluate pH 8, the condition that yielded the best results in the present study [33].

The antiparasitic potential of biologically synthesized AgNPs has been widely documented, having been tested against vectors, protozoa, ectoparasites, and nematodes [14]. Previous studies assessing the anthelmintic activity of biofabricated AgNPs on *H. contortus* eggs and adults [37] reported reduction percentages comparable to those observed in our study. The AgNPs produced by those authors ranged from 28 to 44 nm—similar to the DLS-measured size obtained here (57.9 nm). According to their findings, nanoparticle size was critical for achieving a low LC50 (0.007 ppm). In egg-hatching inhibition assays, smaller nanoparticles more readily crossed the egg membrane, preventing larval emergence. In adult worms, particle size favored efficient transcuticular absorption, leading to parasite mortality.

The lethal assay was conducted on *H. contortus* L3 larvae. AgNPs showed a notable larval reduction of 91.33% at 125 ppm after 24 h, whereas silver nitrate achieved 83.54% at the same concentration. In contrast, the aqueous extract of *A. cina* produced only 21.54% reduction at 2500 ppm, and ivermectin reached 88.83% at 5000 ppm. The LC_50_ and LC_90_ values for the AgNPs were 4.128 ppm and 17.993 ppm, respectively. [41] reported LC_50_ values of 395.1 ppm for L3 larvae treated with chemically synthesized AgNPs—substantially higher than the value obtained in our study—while their LC_50_ values for eggs and adults were 0.007744 ppm and 3.442 ppm, respectively. These comparisons indicate that green-synthesized AgNPs exhibit superior anthelmintic activity relative to chemically synthesized ones. Overall, AgNPs produced with *A. cina* aqueous extract demonstrated potent activity against L3 larvae, highlighting the need to further evaluate their effects on other life stages, including eggs and adults.

An additional key observation from this study was the potential reduced susceptibility to ivermectin in the *H. contortus* field isolate evaluated. The positive control, ivermectin, tested at a high concentration (0.5 mg/mL), resulted in 88.83% larval mortality at 24 h—substantially lower than the efficacy achieved by the biosynthesized AgNPs at concentrations several orders of magnitude lower. Although preliminary and derived from an in vitro larval mortality assay, this trend is consistent with the escalating problem of anthelmintic resistance reported in global field populations of *H. contortus*, particularly against macrocyclic lactones. The use of ivermectin as a benchmark control remains appropriate for comparative efficacy assessment; however, its relative performance reinforces the urgent need to develop next-generation anthelmintic agents with alternative modes of action. The high activity of *A. cina*-derived AgNPs against this potentially tolerant isolate is therefore highly encouraging, as it indicates that oxidative stress-driven lethality may circumvent established resistance pathways—including drug efflux mediated by P-glycoproteins and mechanisms linked to target-site modification common in macrocyclic lactones such as ivermectin. This capacity to bypass conventional resistance supports the emerging potential of plant-mediated nanoparticle synthesis as a complementary strategy for the control of drug-tolerant nematode strains, advancing a new direction for parasite-selective nanotherapeutic interventions.

Although the mechanism of action of AgNPs against nematodes has not yet been fully elucidated, several studies suggest that AgNPs exert their toxic effects by inducing oxidative stress. This occurs through the increased production of ROS, which can disrupt cellular homeostasis and ultimately trigger cell death [40,41]. Nevertheless, the precise molecular pathways involved remain unclear, underscoring the need for additional research to better define how AgNPs exert their anthelmintic effects. One of the defense mechanisms described in *H. contortus* involves the production of antioxidant enzymes that counteract the harmful effects of ROS. Among the main components of this system are SOD, CAT, and GPx, which enable the parasite to mitigate oxidative damage generated by host metabolites, enzymes, or immune proteins [26,32]. Relative gene expression assays are widely used to quantify these stress biomarkers in L3 larvae of ruminants and have previously served as valuable tools for assessing parasitic resistance to various anthelmintics in *H. contortus* L3 populations [32].

The oxidative stress–related genes SOD, GPx, and CAT constitute a key defense system in several ruminant pathogens, including the hematophagous nematode *H. contortus*. These enzymes help neutralize reactive oxygen species generated during host–parasite interactions or exposure to external stressors. Compounds present in *A. cina* have demonstrated toxicity against multiple developmental stages of *H. contortus*—both in vitro and in vivo—when tested as crude extracts under experimental conditions [35].

Given its efficacy of approximately 90%, *A. cina* has been considered a promising candidate for optimization through improved formulation and delivery strategies. The working concentration of the nanoparticles was initially selected based on the previously determined LC_50_ value (4.128 ppm). Gene expression was first evaluated at a low dose (3.9 ppm) over a 24 h period; however, no measurable activation of ROS-related genes was detected, likely due to the extensive cellular damage caused by the nanoparticles at that concentration and exposure duration. Consequently, additional dilutions were tested, leading to the identification of 0.48 ppm as the optimal dose. At this concentration, high-quality total RNA was obtained, allowing reliable assessment of oxidative stress gene expression. These findings indicate that lower nanoparticle doses are necessary for gene expression studies in *H. contortus* L3 larvae. Exposure to AgNPs resulted in a rapid oxidative stress response in *H. contortus* L3 larvae. At 30 min, SOD expression increased while GPx and CAT remained at baseline levels. By 2 h, all three genes (SOD, GPx, and CAT) were upregulated, indicating a coordinated antioxidant response. At 4 h, SOD expression declined to near-constitutive levels and remained normalized at 8 h, whereas CAT showed a notable increase at this later time point. These findings indicate that SOD responds quickly to AgNPs-induced stress (showing early overexpression at 30 min) followed by a decline as exposure progresses. This early and transient activation highlights SOD’s central role in initiating the antioxidant defense triggered by the combined action of *A. cina* metabolites and AgNPs on L3 larvae. The subsequent modulation of SOD expression suggests a regulatory mechanism in response to nanoparticle-induced oxidative stress, supporting its relevance as a biomarker for assessing such cellular damage [32].The first line of antioxidant defense is SOD, which catalyzes the dismutation of the superoxide radical into molecular oxygen and hydrogen peroxide (2O_2_^−^ + 2H^+^ → H_2_O_2_ + O_2_) through alternating reduction and oxidation of its active-site metal ions [41]. Our findings indicate that at a low concentration of AgNPs (0.48 ppm), SOD expression is stimulated, reflecting an induced antioxidant response. Similar observations showed that high concentrations of ZnO nanoparticles suppress antioxidant enzyme production, whereas low concentrations increase ROS levels and thereby stimulate antioxidant enzyme activity to counteract oxidative stress [41]. A comparable phenomenon may explain the results obtained in the lethal assay: higher concentrations of AgNPs produced approximately 90% mortality in *H. contortus*, likely because excessive ROS generation at these concentrations overwhelmed the larvae’s detoxification systems. Such inhibition may occur due to enzyme saturation caused by an overproduction of hydroxyl radicals and ROS, rendering the antioxidant defense machinery ineffective [41].

In the present study, GPx activity decreased at 2 h but returned to baseline levels at 4 and 8 h. This early reduction may be associated with the rapid degradation of antioxidant enzymes under oxidative pressure [40]. Similar findings have been reported in studies using copper and ZnO nanoparticles, where decreased GPx and glutathione-S-transferase (GST) activities accompanied increased ROS levels in the trematode *Fasciola hepatica*, ultimately compromising the parasite’s ability to withstand host-induced oxidative stress [40]. GPx plays a central role in maintaining cellular redox balance by catalyzing the removal of ROS; therefore, reduced GPx expression disrupts redox homeostasis and weakens the parasite’s capacity to neutralize free radicals [39]. Additionally, during oxidative stress, GPx-related enzymes consume glutathione to detoxify hydrogen peroxide by reducing it to alcohols. Excessive ROS generation can deplete glutathione, limiting its availability as a substrate and further diminishing GPx activity [38]. Catalase (CAT) acts downstream of SOD by converting hydrogen peroxide into water and oxygen. This enzyme plays a critical protective role against peroxide in *H. contortus*. Kotze & McClure [1] demonstrated that inhibiting CAT activity in *H. contortus* using aminotriazole increased the nematode’s susceptibility to hydrogen peroxide. Additionally, Kotze [1] reported that both adult worms and the first endoparasitic larval stage (L4) exhibited CAT activity after exposure to low levels of hydrogen peroxide, and that the higher CAT activity observed in adults was associated with an enhanced ability to withstand toxic peroxide levels compared with controls not previously exposed. In the present study, CAT activity decreased at 2 h, normalized at 4 h, and increased again at 8 h. This pattern suggests that CAT may be activated over longer exposure periods relative to SOD. A study by Zelck et al. [2] reported that stimulation with hydrogen peroxide increased the expression of SOD and GPx in *Schistosoma* larvae (a trematode affecting humans), whereas prolonged exposure inhibited SOD expression. In our study, exposure of L3 larvae to 0.078% hydrogen peroxide resulted in the subexpression of all genes at 30 min and 4 h. However, SOD was overexpressed at 2 h and then downregulated again at 4 and 8 h, a pattern consistent with that described by Zelck and colleagues. It is important to note that SOD generates another ROS (hydrogen peroxide) which is uncharged and therefore diffuses freely across cell membranes. Hydrogen peroxide is known to be highly toxic to helminths [2]. In the present study, the aqueous extract of *A. cina* at 2500 ppm increased CAT expression at 30 min, while SOD was overexpressed at 2 h and remained elevated at 4 h, together with GPx. By 8 h, all genes showed downregulation. An increase in ROS levels has been associated with a high content of phenolic compounds in aqueous plant extracts, as polyphenols can act either as antioxidants or pro-oxidants depending on their concentration, pH, and the cellular environment [1,2]. Our findings suggest that *H. contortus* initially activated its antioxidant defense system to detoxify the free radicals induced by the aqueous extract, increasing the expression of antioxidant enzymes and subsequently reducing their activity once the oxidative challenge diminished. It has been well documented that ROS can either induce or suppress gene expression, and that excessive ROS production may not only cause irreversible damage to cellular macromolecules but also repress gene transcription [41].

## 5. Conclusions

The aqueous extract of *A. cina* facilitates the reduction of silver ions and simultaneously acts as a stabilizing agent in the synthesis of AgNPs, producing spherical and notably stable particles (average size: 7.56 nm by TEM and 57.9 nm by DLS). These biogenic AgNPs demonstrated significant anthelmintic activity against *H. contortus* L3 larvae, likely through the induction of oxidative stress that disrupts the parasite’s antioxidant defense system during prolonged exposure. Although the results underscore their potential as sustainable alternatives in the context of increasing anthelmintic resistance, further research is required to assess their efficacy against other developmental stages (e.g., eggs, adults), their broader larvicidal activity, cytotoxicity in animal models, and in vivo performance to validate their practical use. Overall, this study highlights the promise of biologically synthesized AgNPs for parasite control, while emphasizing the need for comprehensive safety and efficacy evaluations prior to field implementation.

## Figures and Tables

**Table 1:** Primer sequences of ARN of oxidative stress genes of *Haemonchus contortus.*

Gene	Access Number	Sequences	pb
Glutathione peroxidase	GQ927327.1	FW- 5′ACGTCGCATCTCAATGTGGT 3′ RV- 5′TGGCGTAGAGATCAGGCTCA 3′	195
Catalase	Maza-López et al. [33]	FW- 5′GAGACGTTGCTCGACATGAA 3′ RV- 5′TTCTGAATCGCTCGATCTTGT 3′	163
Dismutase super oxide	Maza-López et al. [33]	FW- 5′AAAGGCGAAATCAAGGGTTT 3′ RV- 5′TGCTACACCATCAGCTCCAG 3′	192
18S ribosomal ARN	KC998764.1	FW- 5′AGGACTGCGGACTGCTGTAT 3′ RV- 5′AGAGCTTTAACGGGGGTGAT 3′	228

**Table 2:** In vitro larvicidal effect of silver nanoparticles (AgNPs) synthesized using *A. cina* aqueous extract against *H. contortus.*

Treatment	Concentration (ppm)	Reduction (%) ± 24 h
AgNPs	500	91.33 ± (0) ^a^
	250	91.33 ± (0) ^a^
	125	91.33 ± (0) ^a,b^
	62.5	90.145 ± (0.79) ^a,b,c^
	31.25	83.9 ± (2.17) ^b,c,d^
	15.6	77.16 ± (1.78) ^c,e^
	7.8	75.29 ± (1.93) ^e^
	3.9	34.38 ± (4.92) ^f^
	1.95	10.62 ± (2.03) ^h^
Ivermectin	5000	88.83 ± (2.41) ^a,b,c^
AgNO_3_ solution	500	83.54 ± (1.7) ^d,c^
Aqueous extract of *A. cina*	2500	21.54 ± (7. 09) ^g^
Distilled water	-	8.68 ± (1.54) ^h^

Different letters show statistical differences.

**Table 3:** Fold-change and *p*-value results for GPx, SOD, and CAT gene expression at 30 min, 2, 4, and 8 h post-exposure to hydrogen peroxide.

Hydrogen Peroxide (0.078%)								
	30 min		2 h		4 h		8 h	
Gene	Fold-Change	*p*-Value	Fold-Change	*p*-Value	Fold-Change	*p*-Value	Fold-Change	*p*-Value
**SOD**	0.03	0.000039 *	**49.64**	0 *	0	0.001046 *	1.99	0.03245 *
**GPx**	0	0.001238 *	0.79	0.703871	0.02	0.00366 *	0.91	0.424886
**CAT**	0.29	0.003898 *	0.16	0.064814	0.01	0.012047 *	0.23	0.003815 *
**18s**	1	0	1	0	1	0	1	0

**bold numbers**: upregulation; underlined number: downregulation; * significant values (*p* < 0.05). SOD: Superoxide dismutase, GPx: Glutathione peroxidase, CAT: catalase.

**Table 4:** Fold-change and *p*-value results for GPx, SOD, and CAT gene expression at 30 min, 2, 4, and 8 h post-exposure to AgNO_3_.

AgNO_3_ 0.48 ppm								
Gene	30 min		2 h		4 h		8 h	
	Fold-Change	*p*-Value	Fold-Change	*p*-Value	Fold-Change	*p*-Value	Fold-Change	*p*-Value
**SOD**	**2**	0.044309 *	**2.9**	0.515742	0	0.001039 *	0.18	0.051553
**GPx**	0.09	0.002222 *	0.09	0.010241 *	0.01	0.003464 *	1.1	0.84151
**CAT**	**9.68**	0.000103 *	0.04	0.040584 *	0.04	0.014027 *	0.12	0.002992 *
**18s**	1	0	1	0	1	0	1	0

**bold numbers**: upregulation; underlined number: downregulation; * significant values (*p* < 0.05). SOD: Superoxide dismutase, GPx: Glutathione peroxidase, CAT: catalase.

**Table 5:** Fold-change and *p*-value results for GPx, SOD, and CAT gene expression at 30 min, 2, 4, and 8 h post-exposure to *Artemisia* cina aqueous extract.

*Artemisia cina* Aqueous Extract (2500 ppm)								
Gene	30 min		2 h		4 h		8 h	
	Fold-Change	*p*-Value	Fold-Change	*p*-Value	Fold-Change	*p*-Value	Fold-Change	*p*-Value
**SOD**	1.45	0.265849	**26.91**	0.133419	**2.24**	0.106155	0	0.000906 *
**GPx**	0.9	0.783023	0	0.005919 *	**3.33**	0.070151	0.02	0.034336 *
**CAT**	**3.02**	0.000509 *	0	0.02662 *	0.67	0.626506	0.01	0.000369 *
**18s**	1	0	1	0	1	0	1	0

**bold numbers**: upregulation; underlined number: downregulation; * significant values (*p* < 0.05). SOD: Superoxide dismutase, GPx: Glutathione peroxidase, CAT: catalase.

**Table 6:** Fold-change and *p*-value results for GPx, SOD, and CAT gene expression at 30 min, 2, 4, and 8 h post-exposure to AgNPs synthesized using the aqueous extract of *A. cina*.

AgNPs (0.48 ppm)								
	30 min		2 h		4 h		8 h	
Gene	Fold-Change	*p*-Value	Fold-Change	*p*-Value	Fold-Change	*p*-Value	Fold-Change	*p*-Value
**SOD**	**4.31**	0.180169	0.21	0.931112	0.17	0.141545	**2.67**	0.012300 *
**GPx**	0.74	0.924563	0.13	0.025614 *	0.66	0.228491	0.76	0.733067
**CAT**	0.77	0.526789	0.09	0.041339 *	0.62	0.620061	0.43	0.277101
**18s**	1	0	1	0	1	0	1	0

**bold numbers**: upregulation; underlined number: downregulation; * significant values (*p* < 0.05). SOD: Superoxide dismutase, GPx: Glutathione peroxidase, CAT: catalase.

## Data Availability

The raw data will be available upon request from the reader.
